# Predicting the Efficacy of Novel Synthetic Compounds in the Treatment of Osteosarcoma *via* Anti-Receptor Activator of Nuclear Factor-κB Ligand (RANKL)/Receptor Activator of Nuclear Factor-κB (RANK) Targets

**DOI:** 10.2174/0115734064287922240222115200

**Published:** 2024-03-11

**Authors:** Wenhua Zhang, Siping Xu, Peng Liu, Xusheng Li, Xinyuan Yu, Bing Kang

**Affiliations:** 1 The First Clinical Medical College of Gansu University of Chinese Medicine, Gansu University of Traditional Chinese Medicine, Gansu, China;; 2 Department of Plastic Surgery, The Second Clinical College of Lanzhou University, Gansu, China;; 3 The 940^th^ Hospital of Joint Logistics Support force of Chinese People's Liberation Army, Gansu, China

**Keywords:** Osteosarcoma, tumor targeting agents, QSAR, GEP, drug design, HM algorithm

## Abstract

**Background:**

Osteosarcoma (OS) currently demonstrates a rising incidence, ranking as the predominant primary malignant tumor in the adolescent demographic. Notwithstanding this trend, the pharmaceutical landscape lacks therapeutic agents that deliver satisfactory efficacy against OS.

**Objective:**

This study aimed to authenticate the outcomes of prior research employing the HM and GEP algorithms, endeavoring to expedite the formulation of efficacious therapeutics for osteosarcoma.

**Methods:**

A robust quantitative constitutive relationship model was engineered to prognosticate the IC_50_ values of innovative synthetic compounds, harnessing the power of gene expression programming. A total of 39 natural products underwent optimization *via* heuristic methodologies within the CODESSA software, resulting in the establishment of a linear model. Subsequent to this phase, a mere quintet of descriptors was curated for the generation of non-linear models through gene expression programming.

**Results:**

The squared correlation coefficients and *s*2 values derived from the heuristics stood at 0.5516 and 0.0195, respectively. Gene expression programming yielded squared correlation coefficients and mean square errors for the training set at 0.78 and 0.0085, respectively. For the test set, these values were determined to be 0.71 and 0.0121, respectively. The *s*2 of the heuristics for the training set was discerned to be 0.0085.

**Conclusion:**

The analytic scrutiny of both algorithms underscores their commendable reliability in forecasting the efficacy of nascent compounds. A juxtaposition based on correlation coefficients elucidates that the GEP algorithm exhibits superior predictive prowess relative to the HM algorithm for novel synthetic compounds.

## 1 INTRODUCTION

Osteosarcoma (OS) is fundamentally characterized as a malignancy of Mesenchymal Stem Cells (MSCs) that initiates from the formation of the bone matrix [1, 2]. It predominantly manifests in the long bones, with the humerus accounting for approximately 10%, the tibia around 24%, and the femur approximately 52%. Notably, the tumor site frequently presents with symptoms, such as pain, swelling, impaired joint mobility, and skeletal disturbances, with a notable predisposition towards lung metastasis [3]. Alarmingly, only 60% to 70% of patients have been found to achieve a 5-year survival rate, with a marked predominance in males. Metastatic or recurrent cases exhibit a sobering overall survival rate lingering at 25%.

Presently, our understanding postulates that the pathogenesis of osteosarcoma is underpinned by aberrant regulation of mesenchymal cells, anomalies in tumor suppressor gene differentiation, oncogene activation, epigenetic perturbations, and cytokine synthesis. Such processes catalyze the proliferation and multifaceted differentiation of cells, orchestrated by intricate signaling pathways and a plethora of regulatory factors [4]. Osteosarcoma's therapeutic landscape is expansive, encapsulating modalities ranging from surgical interventions and chemotherapy to innovative strategies, like molecularly targeted therapy, immunotherapy, gene therapy, embolization, radiofrequency ablation, and stem cell therapy. Nonetheless, it is disconcerting that, despite these advancements, the therapeutic efficacy has remained stagnant for the past three decades [5], accentuating the imperative for novel, potent therapeutic agents.

Grounded in the understanding that osteosarcoma's genesis is intricately linked to osteoblasts [6, 7], it is worth noting that the nuclear factor κb ligand-receptor activator (RANKL), an osteoblast secretory product, has been classified within the Tumor Necrosis Factor (TNF) receptor ligand family [8-11]. This molecule avidly binds to the nuclear factor κb receptor (RANK) present on osteoblasts, subsequently fostering their maturation, differentiation, and heightened functional activity. Concurrently, the secretion of Osteoprotegerin (OPG) by osteoblasts plays a pivotal role in osteoclast dynamics, as OPG competitively binds to RANKL, effectively curtailing osteoclast activity. Presently, the RANKL/RANK/OPG axis is lauded as a cardinal mechanism governing bone metabolism [12]. Delving into the RANKL/RANK signaling cascade, it emerges that osteosarcoma cells amplify RANKL expression *via* the secretion of parathyroid hormone-related protein (PTHrP). This upregulation bolsters osteoclast activity, and the subsequent liberation of growth factors during cellular lysis amplifies tumor cell proliferation and PTHrP synthesis, culminating in a detrimental feedback loop instigating lytic bone lesions. Evidently, this signaling cascade offers a promising therapeutic target for osteosarcoma. Notably, a slew of investigations have honed in on this potential, elucidating agents such as RANKL inhibitors (which thwart osteoclastogenesis by suppressing RANKL activity) and non-peptide organic moieties (which deter osteoclastogenesis by competitively binding to RANKL) [13]. Yet, these agents are marred by pronounced side effects, underscoring the pressing need for novel drug entities.

In a groundbreaking endeavor, Jiang and Peng [13] unveiled a suite of innovative RANKL/RANK inhibitors. Their seminal work spotlighted a compound (referred to as “1”) showcasing marked affinity for RANKL and RANK proteins, as identified through virtual screening. Subsequent Surface Plasmon Resonance (SPR) assessments and Rankl-driven osteoclastogenesis assays have attested to the compound's binding prowess and its robust osteoclastogenic inhibitory capacities. This revelation catalyzed the synthesis of 39 analogous compounds, among which compounds 2, 7, 29, and 34 emerged as formidable inhibitors of osteoclast differentiation, while demonstrating minimal cytotoxicity. Moreover, rigorous molecular assays utilizing RT-PCR and WB techniques substantiated that compound 34 notably suppressed the expressions of NFATc1 and c-FOS signaling pathways, which are downstream of the RANKL/RANK nexus, thereby inhibiting the excretion of bone-resorbing enzymes.

To augment our insights into the drug's therapeutic potential, we have leveraged computational paradigms for molecular simulation and drug design. This modern approach eclipses traditional drug discovery modalities in terms of cost-effectiveness and efficiency. Historically, Quantitative Structure-Activity Relationships (QSAR) have been instrumental in dissecting databases, deciphering the quantitative interplay between a molecule's structural intricacies and its experimental attributes. Through adept QSAR modeling, we aim to develop novel, high-efficacy drugs for expeditious experimental deployment [14].

## 2 METHODS

Within the confines of this study, a compilation of 39 avant-garde RANKL inhibitor architectures alongside their respective IC_50_ values was examined [13] (Table **1**). A subset comprising thirty molecular structures was judiciously segregated to create the model, while the remaining nine were earmarked for the creation of the validation or test set.

### 2.1 Calculation of Descriptors

At the forefront of constructing a QSAR modellies the quintessential step of utilizing descriptors to epitomize compounds. Our initial maneuver involved channeling the 39 structures into the ChemDraw platform. A cohort of thirty molecules was chosen at random for the training ensemble, with the subsequent nine designated as the testing cluster. These delineated structures were then integrated into the HyperChem software suite [15, 16], whereupon the MM+ molecular mechanics force field, coupled with the semi-empirical AM1 or PM3 methodologies [17], was harnessed for optimal structural refinement. Culminating this phase, intricate computations were orchestrated *via* the MOPAC 6.0 software, yielding three distinct files for every individual structure.

### 2.2 Linear Modeling with Heuristics

For transitioning the MNO files into the CODESSA computational environment [18], the discerning selection of parametric variables emerged as pivotal. This rigorous exercise bore 551 descriptors in total. Employing Heuristic Methodologies (HM) proved invaluable for descriptor sieving [15, 19]. Certain benchmarks were imperative [20]: (a) extirpation of null or “0” variables; (b) culling of overly-correlated variables precipitating suboptimal outcomes; and (c) the eradication of variables beset with lackluster correlations amid attributes and said variables. *via* the HM paradigm, a select quintet of descriptors was crystallized for the synthesis of the superlative multiple linear regression model. These descriptors can be taxonomically classified into five dominant categories: constitutive, topological, geometric, electrostatic, and quantum chemical descriptors. The entire operational methodology is predicated on the sequential augmentation of descriptors, with precision anchored by paramount regression coefficients, namely the R^2 value, cross-validated R^2 (R^2cv), and the F-statistic (F) value. While R^2cv assays reliability, the triumvirate of R^2, F, and s^2 vouches for model authenticity. The heuristic-centric optimization algorithm is lauded for its lucidity, rapidity, and determinate computational duration. Notably, the intrinsic efficiency of HM, particularly its temporal economy, underscores its preference over alternative methodologies [18].

### 2.3 Nonlinear Modeling of the GEP Approach

Gene Expression Programming (GEP) emerges as an evolutionary algorithm, intrinsically inspired by the intricate nuances of biological gene architecture and functionality [21]. The foundational tenets of the GEP algorithm encompass the stochastic generation of a specified quantum of chromosomal entities (dubbed the initial populace). Subsequently, these chromosomes undergo expression, post which the fitness of every singular entity is assayed against a predetermined fitness sample suite (the problem's input criterion). Culminating this, predicated on the ascertained fitness metrics, entities are earmarked for genetic modification, spawning progeny endowed with novel traits. This cyclical process perpetuates across myriad generations, culminating in the identification of an optimal solution. The GEP algorithmic framework is demarcated into quintessential stages: delineation of the coding schema, the fitness function, and the operational function set (+, -, *, /); calibration of control parameters; meticulous crafting of the network operator; and the rigorous establishment of terminal benchmarks [22].

## 3 RESULTS

### 3.1 HM Calculations

Through the employment of the CODESSA software suite, 39 molecular compounds were meticulously computed, resulting in the generation of a total of 551 descriptors. An intriguing trend was observed, wherein, with an ascending quantity of descriptors, there was a concurrent increment in the values of R^2 and R^2cv, while s^2 exhibited a persistent decrement. The fluctuations in these values became negligible when the number of parameters approached 2, a point aptly termed the “breaking point” [23, 24]. Concurrently, this juncture also satisfied the conditions set by the number of parameters (k) and sample size (n): n ≥ 3 (k+1). Fig. (**1**) lucidly portrays this empirically derived data. Consequently, a linear QSAR model was materialized wherein the robustness of R^2 and F vouched for the model's validity, and R^2cv served as a testament to its stability. The intricate specifics of these parameters are illuminated in Fig. (**1**).

### 3.2 Results of GEP Calculations

In a bid to juxtapose the predictive acumen of HM against GEP, the twin descriptors delineated by the HM protocol were transposed into the APS software infrastructure [20], culminating in the derivation of a nonlinear model. This task was bifurcated into distinct phases: initially, the dataset was stochastically partitioned into a testing ensemble (comprising 30 compounds) and a training cadre (consisting of 9 compounds). Subsequently, the model was engendered by processing the training data, with its reliability vetted against the test set. A tabulation of the pertinent parameters is presented in Table **1**. For both the training and testing sets, the R^2 values were discerned as 0.78 and 0.71 respectively, with corresponding mean squared errors being 0.0085 and 0.0121. The equations for the nonlinear model, as manifested within the expression tree, followed suit.

### 3.3 Comparison of GEP and HM Algorithms

**Fig. (2):**
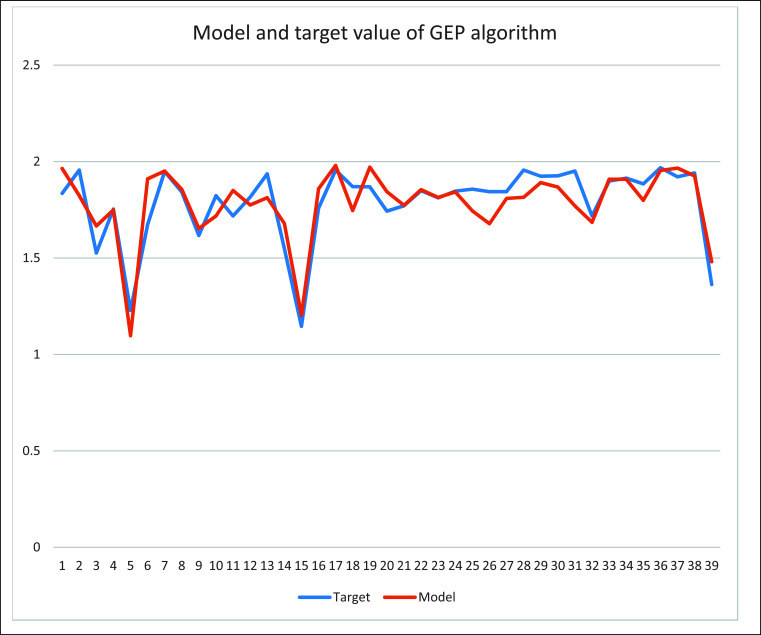
Comparison of linear and nonlinear models.

As evident in Fig. (**2**), the correlation coefficients deduced *via* the GEP algorithm consistently eclipsed those of the HM algorithm within the training subset. Furthermore, the error margins associated with GEP were discernibly more diminutive in comparison to the HM algorithm. This observation held consistent validity not just within the training subset, but also mirrored it in the testing set, wherein the GEP algorithm's error magnitude substantially outperformed that of the HM methodology (Table **1** and Fig. **2**). This empirical juxtaposition underscores the formidable modeling prowess of GEP alongside its commendable generalization capabilities, reinforcing its potential as a trustworthy instrument for the prediction of IC_50_ values pertinent to pharmaceutical compounds.

## 4 DISCUSSION

For a nuanced understanding of the impact of each descriptor parameter on the IC_50_ value, a thorough examination of each descriptor is imperative. This meticulous analysis can help facilitate the extraction of critical physicochemical information influencing the IC_50_ value, thereby providing invaluable insights for the development of more potent compounds. Within the framework of the HM model, the molecular geometry is intrinsically determined by the number of backbone atoms. In the context of this study, carbon atoms constitute the primary backbone affecting the semi-inhibition rate. Importantly, the architectures crafted by various arrangements of carbon atoms wield a discernable influence on the IC_50_ value. To illustrate, the molecular structure of methyl imidazole, enriched by an additional methyl group as compared to pyridine, fortifies the lipophilic attributes of the compound. Ionic liquids exhibiting elevated degrees of lipophilicity are consequently more readily adsorbed and aggregated within the cellular membranes of biological entities. Moreover, a direct positive correlation exists between the descriptor KSI and the IC_50_ value, signifying an amplification in the toxicity of ionic liquids concomitant with an increase in the descriptor value. RNCS serves as an illustrative descriptor demarcating the spatial distribution of negative charges within ionic liquids, and its significance is inextricably linked to hydrogen bonding capabilities. Given that bacterial membranes inherently possess a negative charge, an augmentation in the negatively charged surface area of ionic liquids induces electrostatic repulsion, thereby inhibiting effective membrane binding. Such alterations in membrane conformation are instrumental in conferring toxicity, as substantiated by extant literature [16]. Consequently, in the quest for engineering environmentally benign ionic liquids, novel structures exhibiting reduced toxicity could be realized through judicious modulation of the KSI and RNCS values. The descriptor RNDB quantifies the relative presence of double bonds in ionic liquids and is categorized as a compositional descriptor. The incorporation of double bonds within either the cationic (*e.g*., c=c) or anionic (*e.g*., -s=o) backbone considerably influences the binding affinity of the ionic liquid to the target. Additionally, oxidative cleavage or reduction of these double bonds has the potential to modulate the compound's toxicity profile [17-18]. Therefore, RNDB's role as a pivotal model parameter is accentuated by its pronounced impact on the pEC_50_ value, revealing a negative correlation with the same.

The inclusion of the descriptor RNDB in the model parameterization is of paramount importance, particularly owing to its pronounced influence on the pEC_50_ value associated with Vibrio cyanobacteria Q67. Notably, RNDB manifests a negative correlation with the pEC_50_ value, underscoring its salient role in the toxicity modulation of ionic liquids.

The descriptor NFA delineates the number of fluorine atoms incorporated into the ionic liquid and has a significant bearing on the toxicity attributes of the compound. Ionic liquids typically employ carbon atoms as their skeletal backbone and synergize with hydrogen to form hydrocarbon chains. The introduction of fluorine atoms, either partially or completely supplanting hydrogen atoms, culminates in the formation of fluorocarbon surfactants, characterized by fluorine-dominated chains [15]. Given that fluorine is the most electronegative element, and the fluorine-carbon bond boasts the highest bond energy among covalent bonds, fluorocarbon structures exhibit elevated stability compared to their hydrocarbon counterparts [19]. In the milieu of biological metabolic oxidative decomposition, fluorine-substituted ionic liquids demonstrate superior stability. Additionally, fluorocarbon surfactants possess exceptional compatibility properties, facilitating the diffusion and absorption of ionic liquids into biological entities, thereby enhancing toxicity. These observations collectively elucidate the positive correlation between NFA and pEC_50_ values in the HM model; specifically, an increase in fluorine atom count amplifies the inhibitory efficacy of ionic liquids against bioluminescent bacteria.

The descriptor ABOCA is an acronym representing the Average Bonding modality of Carbon Atoms within the ionic liquid. The bonding modes, whether single, double, or triple, between carbon atoms, can vary substantially among different ionic liquids, significantly impacting the ABOCA value. The susceptibility of double and triple bonds to oxidation contributes to toxicological effects, including perturbations in cellular integrity and potential genetic material modifications [20]. Literature corroborates the notion that the alkyl chain length in ionic liquids exerts a significant influence on their toxicity. An increase in alkyl chain length augments the lipophilicity of the ionic liquid, thereby enhancing its adsorptive and aggregative propensity on biological cellular membranes. Such a phenomenon facilitates the disintegration of the cellular membrane architecture, culminating in the eradication of the biological entity and a consequent reduction in luminescence intensity. This mechanistic understanding substantiates the role of ABOCA in increasing the pEC_50_ value within the HM model. In summary, modulating the lipophilicity of an ionic liquid could serve as a viable strategy for attenuating its inherent toxicity.

This investigation presents a robust Comparative Molecular Similarity Indices Analysis (COMSIA) model, distinguished by noteworthy statistical parameters, including a high cross-validated correlation coefficient (q^2) of 0.529, a non-cross-validated correlation coefficient (r^2) of 0.993, and a minimal Standard Estimated Error (SEE) of 0.033. Additionally, the COMSIA contour maps elucidate pivotal structural features within steric, electrostatic, hydrophobic, hydrogen bond donor, and hydrogen bond acceptor fields, thereby imparting invaluable predictive insights pertinent to the understanding of structure-activity relationships in pharmacologically active compounds.

The superior predictive power of this model paves the way for rational drug design endeavors. Leveraging this model, 200 novel 1,8-naphthalimide derivatives have been conceptualized, predicated on the structural foundation of compound **7a**. Their IC_50_ values have been prognosticated utilizing the aforementioned COMSIA model. Of these synthetically envisioned entities, the derivative 7a.10 stands out, exhibiting both the most favorable IC_50_ predictive value and optimal docking affinity with the target DNA molecule. Consequently, the findings of this research offer pragmatic directives for the advancement of novel DNA-targeting chemotherapeutic agents specifically engineered for osteosarcoma treatment.

The outcomes of the present study substantiate that both the Harmony Search (HM) and Gene Expression Programming (GEP) algorithms exhibit formidable predictive capabilities. The HM algorithm offers a distinct advantage of computational alacrity, unencumbered by constraints on dataset dimensions or structural format. It is proficient in swiftly generating optimal regression equations, thereby furnishing an array of models from which researchers may judiciously select. Additionally, its automated parameter selection obviates the potential omission of critical descriptors for ionic liquids. Importantly, it delineates not merely the category of descriptor most germane to the activity values, but also ascertains the statistical significance of said descriptor. Conversely, the Gene Expression Programming Algorithm Package (APS) affords a user-friendly modus operandi, enabling iterative model validation *via* test sets, which inherently renders the optimization trajectory more intuitive and facile. This algorithm amalgamates the merits of both genetic algorithms and genetic programming, representing individual entities through fixed-length linear codes, thereby streamlining genetic manipulations and enhancing its prowess in resolving complex problems. Comparative metrics reveal that the R^2 and S^2 values associated with the training sets of the GEP model are demonstrably superior to those of its HM counterpart.

Empirical evidence garnered from the test set substantiates the stability inherent in the Gene Expression Programming (GEP) model, revealing it to marginally outperform the Harmony Search (HM) algorithmic method. Intriguingly, the Quantitative Structure-Activity Relationship (QSAR) model conceived *via* GEP is predicated on a nonlinear function, juxtaposed with the comparatively rudimentary linear function proffered by the HM algorithm [21]. While the incorporation of genetic operations, such as mutation, recombination, and inversion alongside functional manipulations elevates the complexity and operational intricacy of the GEP framework, rigorous optimization techniques yield results of notable satisfaction.

In the ambit of this scholarly investigation, both the HM and GEP algorithms have been judiciously employed to probe the architectural nuances of ionic liquids and their concomitant QSAR with respect to the Q67 toxicity exhibited by Vibrio cyanobacteria. The results yielded two distinct predictive models, of which the toxicity prediction schema engendered by the GEP algorithm demonstrated superior efficacy relative to its HM counterpart.

Successful application of the GEP algorithm to the prediction of ionic liquid activity yielded a QSAR model of enhanced precision. This fortifies the assertion that nonlinear models can be efficaciously deployed in the realm of biological QSAR research, producing results that meet rigorous academic standards. Through meticulous investigation of the involved descriptors, a wealth of information has been gleaned concerning the physicochemical structural elements that govern ionic liquid toxicity. This repository of knowledge may serve to illuminate pathways for the future conceptualization and engineering of ionic liquids with attenuated toxicity profiles.

## CONCLUSION

In the present study, an integrative approach leveraging both Harmony Search (HM) and Gene Expression Programming (GEP) methodologies has been devised to forecast the ligand-binding affinity of pharmaceutical agents with osteosarcoma-associated molecular targets. Employing a rigorous computational framework to ascertain molecular structural descriptors, linear and nonlinear Quantitative Structure-Activity Relationship (QSAR) models have been constructed *via* HM and GEP algorithms. The ensuing predictive outcomes have been adjudged to be commensurate with empirical expectations, thereby affirming the methodological efficacy of the study.

## Figures and Tables

**Fig. (1):**
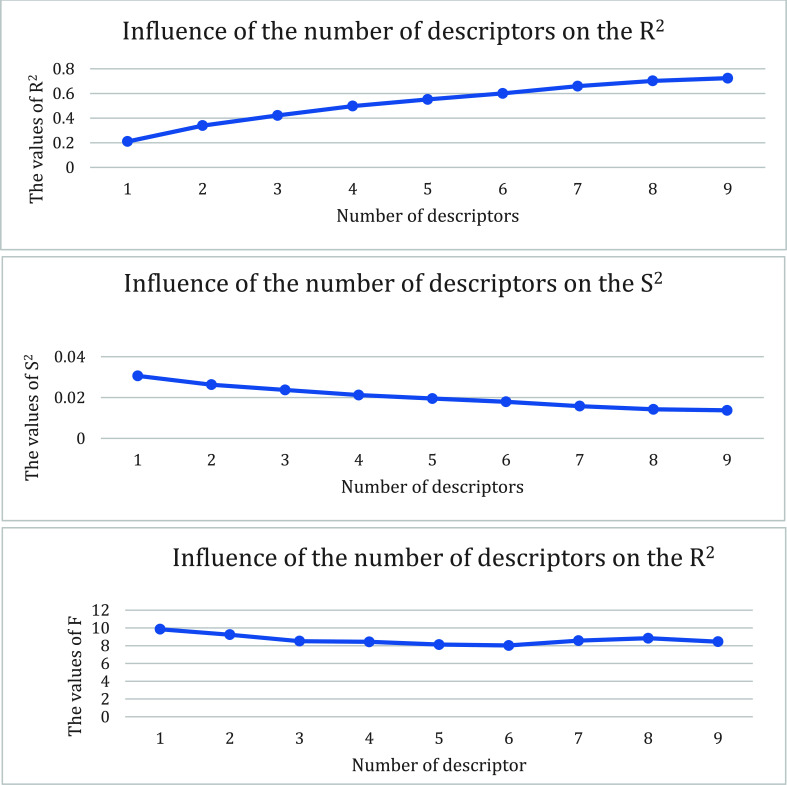
The effects of different numbers of descriptors on R^2^, R^2^cv, and S^2^.

**Table 1:** The experimental and predicted values of the COMSIA model, where the test set data are marked with *.

**Compound**								**TRAP Activities** **(Inhibition,%)**			**HM**						**GEP**				
**Structure**	**No.**	**X**		**R1**							**Predicted**		**Residue**		**Difference**		**Target**		**Model**		**Residual**
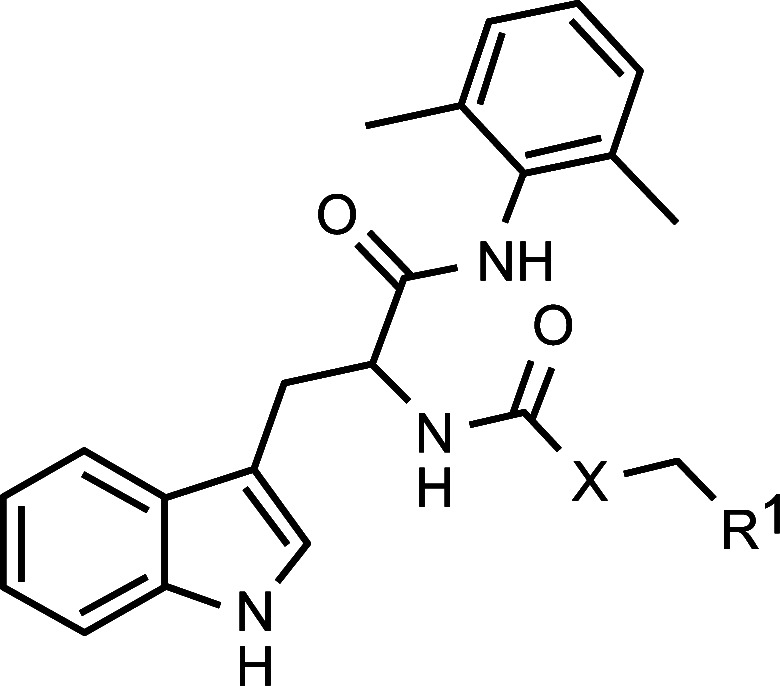	1*	O		Ph				68.4 ± 2.9			1.725		1.835		-0.110		1.835		1.964		0.129
	2	CH_2_		Ph				90.3 ± 0.9			1.763		1.956		-0.192		1.956		1.824		0.131
	3	CH_2_		4-F-phenyl				33.6 ± 1.3			1.770		1.526		0.243		1.526		1.666		0.140
	4	CH_2_		3-F-phenyl				56.8 ± 0.7			1.765		1.754		0.011		1.754		1.749		5.461
	5*	CH_2_		4-OMe-phenyl				16.9 ± 0.1			1.571		1.228		0.343		1.228		1.097		0.130
	6	CH_2_		3-OMe-phenyl				41.7 ± 0.6			1.648		1.673		-0.025		1.673		1.910		0.237
	7	CH_2_		4-Cl-phenyl				89.0 ± 1.5			1.770		1.949		-0.179		1.949		1.951		1.251
	8*	CH_2_		3-Cl-phenyl				69.5 ± 3.2			1.762		1.842		-0.080		1.842		1.856		1.352
	9	CH_2_		4-pyridyl				41.4 ± 1.0			1.705		1.617		0.088		1.617		1.653		0.036
	10*	CH_2_		3-pyridy				66.5 ± 1.1			1.713		1.823		-0.110		1.823		1.718		0.105
	11	CH_2_		3-thienyl				52.2 ± 1.2			1.766		1.718		0.048		1.718		1.850		0.133
	12*	CH_2_		2-thienyl				65.4 ± 1.0			1.763		1.816		-0.053		1.816		1.775		4.100
	13	CH_2_		2-thiazolyl				86.3 ± 2.2			1.678		1.936		-0.258		1.936		1.813		0.123
	14	CH_2_		cyclohexyl				35.4 ± 0.6			1.579		1.549		0.030		1.549		1.679		0.130
	15	CH_2_		cyclopentyl				14.0 ± 0.8			1.567		1.146		0.421		1.146		1.203		5.688
	16*	CH_2_		cyclopropyl				57.4 ± 1.1			1.602		1.759		-0.157		1.759		1.859		0.100
**Compound**								**TRAP Activities** **(Inhibition,%)**			**HM**						**GEP**				
**Structure**	**No.**	**R2**	**R3**			**R4**					**Predicted**		**Residue**		**Difference**		**Target**		**Model**		**Residual**
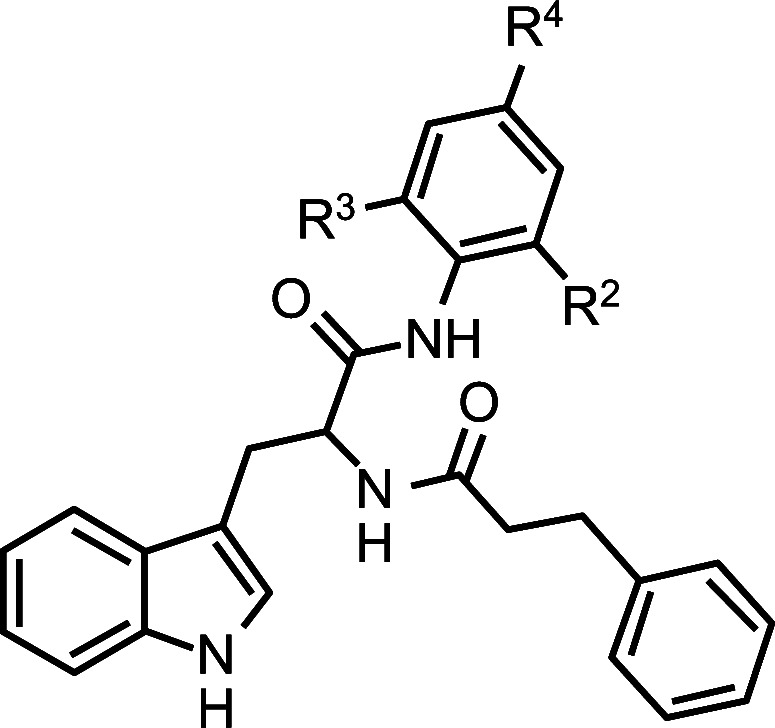	b2	Me	Me			H		90.3 ± 0.9			1.766		1.956		-0.190		1.956		1.980		2.474
	17	Me	H			H		74.2 ± 6.5			1.889		1.870		0.019		1.870		1.746		0.124
	18*	F	H			H		74.0 ± 5.6			1.923		1.869		0.054		1.869		1.971		0.102
	19	H	H			H		55.3 ± 1.8			1.926		1.743		0.183		1.743		1.844		0.102
	20	H	H			Me		69.9 ± 5.4			1.877		1.845		0.033		1.771		1.773		2.267
	21	H	H			OMe		59.0 ± 1.4			1.683		1.771		-0.088		1.849		1.854		4.817
	22	H	H			NMe_2_		70.6 ± 1.9			1.823		1.849		-0.026		1.812		1.814		2.266
	23	H	H			4-morpholinyl		64.8 ± 5.5			1.726		1.812		-0.085		1.847		1.843		4.358
	24	H	H			F		70.3 ± 5.7			1.915		1.847		0.068		1.857		1.744		0.113
	25*	H	H			CF_3_		72.0 ± 3.7			1.875		1.857		0.018		1.844		1.678		0.166
	26	H	H			SO_2_CH_3_		69.9 ± 4.1			1.795		1.845		-0.050		1.844		1.809		3.516
**Compound**										**TRAP ** **Activities** **(Inhibition,%)**		**HM**						**GEP**			
**Structure**	**No.**	**Y**			**Z**		**R5**		**R6**			**Predicted**		**Residue**		**Difference**		**Target**		**Model**	**Residual**
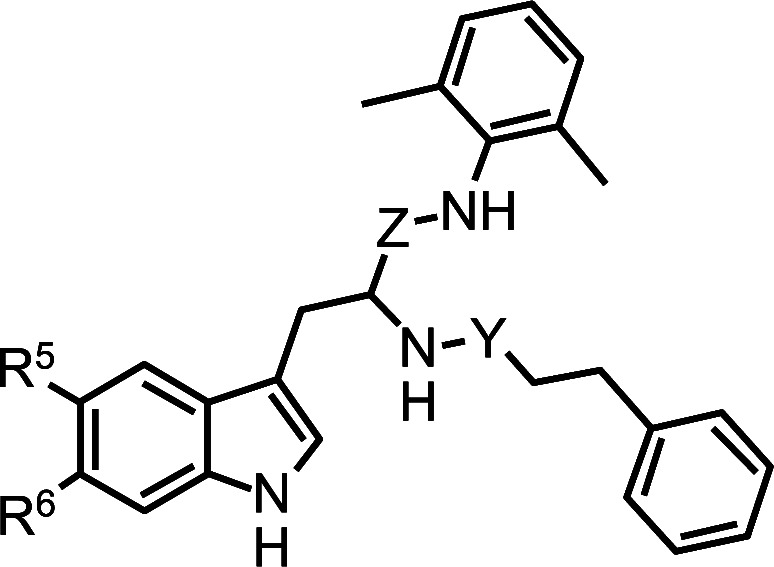	b3	C(=)O			C(=O)		H		H	90.3 ± 0.9		2.002		1.956		0.046		1.956		1.815	0.141
	27	C(=)O			C(=)O		F		H	83.9 ± 2.4		1.980		1.924		0.056		1.924		1.891	3.248
	28	C(=)O			C(=)O		OMe		H	84.4 ± 1.6		1.811		1.926		0.116		1.926		1.868	5.844
	29*	C(=)O			C(=)O		H		H	89.4 ± 1.3		1.966		1.951		0.014		1.951		1.770	0.181
	30	C(=)O			C(=)O		H		H	52.1 ± 1.2		1.890		1.717		0.173		1.717		1.685	3.182
	31	C(=)O			C(=)O		H		H	79.3 ± 0.7		1.916		1.899		0.016		1.899		1.908	8.996
	32	C(=)O			C(=)O		H		H	82.0 ± 3.7		1.869		1.914		-0.045		1.914		1.908	5.725
	33	CH2			C(=)O		H		H	76.5 ± 5.6		1.911		1.884		0.028		1.884		1.799	8.514
	34	CH2			C(=)O		H		H	92.9 ± 1.9		1.985		1.968		0.017		1.968		1.954	1.367
	35	CH2			C(=)O		H		H	83.3 ± 1.6		1.883		1.921		-0.037		1.921		1.966	4.583
	36	CH2			C(=)O		H		H	87.2 ± 0.5		1.833		1.941		-0.108		1.941		1.926	1.487
	37	C(=)O			H		H		H	23.1 ± 1.8		1.364		1.364		0		1.363		1.481	0.117

## Data Availability

The data and supportive information are available within the article.

## LIST OF ABBREVIATIONS

GEP: Gene Expression Programming
QSAR: Quantitative Structure-Activity Relationships
SPR: Surface Plasmon Resonance
OPG: Osteoprotegerin
TNF: Tumor Necrosis Factor
